# Medication-taking behaviour in Bulgarian women with postmenopausal osteoporosis treated with denosumab or monthly oral bisphosphonates

**DOI:** 10.1007/s11657-017-0413-5

**Published:** 2017-12-21

**Authors:** T. Petranova, M. Boyanov, A. Shinkov, R. Petkova, M. Intorcia, E. Psachoulia

**Affiliations:** 1 Clinic of Rheumatology, Department Internal Medicine, University Hospital “St. Ivan Rilsky”, Medical University-Sofia, 13 “Urvich” Str., 1612 Sofia, Bulgaria; 2 Clinic of Endocrinology and Metabolic Diseases, Department Internal Medicine, University Hospital Alexandrovska, Medical University–Sofia, 1 G. Sofiyski Str., 1431 Sofia, Bulgaria; 3 University Hospital of Endocrinology, Medical University–Sofia, 2 Zdrave St, 1574 Sofia, Bulgaria; 4 Amgen Bulgaria EOOD, 63 Kazbek Str., 1680 Sofia, Bulgaria; 5 Amgen (Europe) GmbH, Dammstrasse 23, 6301 Zug, Switzerland

**Keywords:** Bisphosphonates, Bulgaria, Denosumab, Discontinuation, Ibandronate, Osteoporosis, Persistence

## Abstract

**Summary:**

Persistence with osteoporosis therapy is critical for fracture risk reduction. This observational study evaluated medication-taking behaviour of women with postmenopausal osteoporosis receiving denosumab or oral ibandronate in real-world clinical practice in Bulgaria. Compared with ibandronate, densoumab was associated with a lower discontinuation rate and greater increases in bone mineral density.

**Purpose:**

Persistence with osteoporosis therapy is critical for fracture risk reduction and the effectiveness of such treatments may be reduced by low persistence. Alternative therapies such as denosumab may improve persistence. This study aimed to describe medication-taking behaviour in women with osteoporosis, prescribed denosumab or oral ibandronate, in Bulgarian clinical practice.

**Methods:**

This retrospective, observational, multicentre chart review (with up to 24 months follow-up) enrolled postmenopausal women initiating 6-monthly denosumab injection or monthly oral ibandronate treatment for osteoporosis between 1 October 2011 and 30 September 2012.

**Results:**

Overall, 441 women were enrolled (224 had initiated denosumab, 217 had initiated ibandronate). At baseline, more women in the denosumab group than in the ibandronate group had a previous fracture (25.5 vs 17.5%; *p* = 0.043) and past exposure to osteoporosis therapy (19.6 vs 12.0%; *p* = 0.028). At 24 months, 4.5% of women receiving denosumab had discontinued therapy compared with 56.2% of women receiving ibandronate. Median time to discontinuation was longer in the denosumab group (729 days; interquartile range (IQR), 728.3–729.0) than in the ibandronate group (367 days; IQR, 354.0–484.8; *p* < 0.001). At 24 months, there were significantly greater changes in BMD *T*-scores at the lumbar spine (*p* < 0.001) and femoral neck (*p* < 0.001) in patients receiving denosumab than in those receiving ibandronate. At 24 months, persistence with denosumab was 98.7%.

**Conclusion:**

This real-world study demonstrates there is a low discontinuation rate and high persistence with denosumab. Denosumab was associated with greater BMD increases than ibandronate, which could reduce fracture risk.

## Introduction

Osteoporosis is characterised by low bone mineral density (BMD) and disruption of bone architecture, which leads to an increased risk of fractures [1, 2]. The incidence and prevalence of osteoporosis are increasing as a consequence of ageing populations. In Bulgaria, a large epidemiological study by Borissova et al. (2011) found that in women aged 50 years or older, 16.8% had osteoporosis of the femoral neck, and approximately half had BMD *T*-scores at the femoral neck that indicated low bone mass or osteoporosis [3]. A more recent population-based study estimated that approximately 26.6% of Bulgarian women aged 50 years or older have osteoporosis of at least one bone site [4].

A consequence of the increasing prevalence of osteoporosis is that a growing number of women are at risk of fractures, which are associated with considerable pain and disability, as well as substantial healthcare costs [1, 5]. The estimated 10-year absolute fracture risk for women with osteoporosis in Bulgaria is 13.4% for major fractures and 2.8% for hip fractures [3]. This equated to 21,476 fractures among 1.6 million Bulgarian women aged 50 years or older in 2010 [1]. Therefore, osteoporosis is a major public health problem and imposes a huge financial burden [1]. In 2010, the economic burden of osteoporosis in Bulgaria was €42 million. Hip fractures were estimated to be the most expensive type of fracture, accounting for almost half of the economic burden associated with osteoporosis; although hip fractures were not the most frequent fracture type, costs were high at an estimated €1826 for each fracture. Pharmacological fracture prevention accounted for only 3.1% of the total cost [1]. Data on patient outcomes following antiresorptive therapy are required to improve understanding of their value, in terms of osteoporosis treatment and fracture prevention in Bulgaria.

Bisphosphonates, administered orally at daily, weekly or monthly intervals, are commonly used to treat osteoporosis and reduce the risk of fractures [1, 6, 7]. However, persistence and compliance with bisphosphonates are low [8]. Persistence can be defined as the extent to which treatment is continued for the prescribed duration, and compliance can be defined as the extent to which medication is taken correctly as per the prescribed dosage, timing and frequency of treatment. Approximately half of patients discontinue bisphosphonate treatment within 12–24 months [8, 9]. Additionally, over 2 to 5 years, fewer than half of women are compliant with treatment [10–12], and only approximately 20% persist with it [11, 12].

Denosumab, a monoclonal antibody that inhibits RANK-L, is approved for the treatment of osteoporosis in patients with an increased risk of fractures [13]. Treatment with a subcutaneous (SC) injection of denosumab 60 mg every 6 months (Q6M) has been shown to result in a significant reduction in the risk of vertebral, non-vertebral and hip fractures in postmenopausal women [14]. Furthermore, persistence with denosumab has been shown to be high; in retrospective and prospective studies, the proportion of patients persistent at 12 and 24 months ranged from 81 to 95% and 75 to 86%, respectively [15–19]. Also, better medication-taking behaviour has been reported in patients receiving denosumab than in those receiving oral bisphosphonates; in a 24-month randomised, crossover study in which women with osteoporosis received weekly oral alendronate for 12 months followed by denosumab SC Q6M for 12 months (or vice versa), significantly more patients were compliant and persistent with denosumab than with alendronate [20]. Increased persistence and compliance with osteoporosis treatment have been shown to improve BMD and reduce fracture risk [17, 21–23]. A recent retrospective study found that patients who were compliant with osteoporosis medication (defined as a medication possession ratio of ≥ 80%) had a 23% greater reduction in fracture risk compared with patients who were non-compliant [17]. Poor medication-taking behaviour is, therefore, a major barrier to the effective management of osteoporosis.

There are no published data on persistence with denosumab in real-world Bulgarian clinical practice. Such data are critical to understand how therapies affect patient outcomes beyond the clinical trial setting. This study assessed medication-taking behaviour and clinical outcomes among postmenopausal women in Bulgaria who received denosumab or a monthly oral bisphosphonate (ibandronate), and estimated persistence with denosumab, in a real-world setting.

## Materials and methods

### Study objectives

The objective of this study was to describe the pattern of medication-taking behaviour (e.g. time to discontinuation and the number and percentage of patients remaining on therapy) in postmenopausal women receiving denosumab or monthly oral bisphosphonate for up to 24 months. Clinical outcome data (BMD and BMD *T*-scores) at 24 months after treatment initiation were examined for patients receiving either therapy. We also examined the first real-world estimates of 12-, 18- and 24-month persistence with denosumab for Bulgaria.

### Study design

This retrospective, observational, multicentre, chart review study was conducted in 12 endocrinology or rheumatology practices across Bulgaria. The study protocol was approved by a central regulatory ethics committee, in accordance with the ethical principles of the Declaration of Helsinki.

### Participants

Patients were screened according to the eligibility criteria outlined below. In order to obtain as unbiased selection of patients as possible, patients were randomly selected from two lists (one for patients treated with denosumab and one with oral bisphosphonates). For each site, a maximum number of patients was defined and was proportional to the number of patients matching the eligibility criteria in the site.

Women were considered eligible for inclusion if they were aged 50 years or older; had a clinical diagnosis of postmenopausal osteoporosis (defined as BMD ≤ − 2.5 standard deviation (SD) of the mean value for premenopausal women aged 25 years) and had initiated treatment with either denosumab (60 mg SC Q6M) or monthly oral ibandronate between 1 October 2011 and 30 September 2012. Women were not eligible if they were currently participating in, or had previously participated in, a clinical trial for denosumab, or if they had participated in any clinical trial within the previous 6 months.

### Study sites and data collection

The study was conducted at 12 representative endocrinology or rheumatology sites specialising in the treatment of patients with osteoporosis, which were selected from potential sites from across Bulgaria as described previously [24], based on a feasibility assessment of the number of patients with osteoporosis treated, data availability and clinic size. Study participants were selected by site staff from site lists of patients taking either of the prespecified medications within the predefined timeframe. All patients who met the study eligibility criteria were shortlisted for inclusion in the study. If the number of shortlisted patients exceeded the assigned quota of participants for that site, site personnel randomly selected patients from the shortlist to meet the quota required for the study. Data were collected electronically from patient medical records, which included data gathered as part of routine clinical practice during the postmenopausal osteoporosis treatment initiation visit. These data were used to characterise the patient population at baseline. Data relating to denosumab or ibandronate prescription and administration, calcium or vitamin D supplementation and osteoporosis visits were obtained during subsequent patient visits. Patients were followed up for 24 months after treatment initiation.

### Assessment of discontinuation and persistence

Discontinuation was assessed using Kaplan–Meier survival analysis. For both denosumab and ibandronate, the discontinuation date and reason for discontinuation (and additionally any switch to another treatment or dosing regimen) were taken from the patients’ medical records. Time to discontinuation for denosumab and ibandronate was based on the prescription dates and was defined as the time from treatment initiation to the time that patients were no longer covered by medication (i.e. the time point after the last prescription received during the 24-month period plus the prescription coverage period). Moreover, the number and the percentage of the patients still on treatment at 12 and 24 months was calculated. Therefore, the number of patients who had discontinued ibandronate at 12 and 24 months could be calculated; however, persistence with ibandronate could not be calculated owing to a lack of data availability.

Denosumab persistence at 12, 18 and 24 months was defined as receiving the subsequent injection within 6 months plus 60 days (the permissible gap) after the previous injection; sensitivity analysis was performed using 30- and 90-day permissible gaps. The proportion of persistent patients at 12, 18 and 24 months and 95% confidence interval (CI) was analysed. This analysis was performed for each permissible gap (30, 60 and 90 days).

### Assessment of bone mineral density

BMD was measured and BMD *T*-score calculated as part of routine clinical practice during the treatment initiation visit, and at 12 and 24 months following initiation; for each measurement, patients returned to the clinic in which their initial measurement was taken. It is standard clinical procedure that each measurement is taken on the same dual-energy X-ray absorptiometry machine. The mean change in BMD and BMD T-score from baseline to 24 months of follow-up was calculated for all patients who initiated therapy and who had BMD and BMD *T*-score measurements from both time points (baseline and 24 months).

### Statistical analyses

All demographic and clinical characteristics were summarised using descriptive analyses. Categorical variables were summarised using the number, percentage and 95% CI. Depending on the size of the category, *χ* ^2^ values were calculated with or without continuity correction or using a Monte Carlo simulation. Continuous variables were summarised using the mean ± SD, median, minimum, maximum and 95% CI. For all continuous variables, Student’s *t* test, Welch’s test or Wilcoxon’s test was conducted. The *F*-test was used to check the equality of variance assumptions, and the Shapiro–Wilk test was used to check the normality of distribution assumptions. Median time to non-persistence (Q1, Q3) was reported. Time to non-persistence was analysed using Kaplan-Meier curves for the denosumab group over time. A multivariate Cox model was used to obtain the hazard ratio for discontinuation (denosumab vs ibandronate). The following covariates were considered: age, previous fragility fracture, previous postmenopausal osteoporotic therapy, calcium supplementations, vitamin D supplementations and systemic glucocorticoid use. A paired two-sample Student’s *t* test was conducted to assess changes in BMD and BMD *T*-score from baseline to 24 months for patients receiving denosumab and for those receiving ibandronate. A significance threshold of *p ≤* 0.05 was applied to all statistical tests.

## Results

### Patient characteristics

In total, 441 women who met the eligibility criteria were included in this chart review from 12 sites in Bulgaria. This included 224 women who had initiated treatment with denosumab and 217 who had initiated treatment with ibandronate. The baseline patient characteristics are summarised in Table 1. Mean age at the time of enrolment was similar in the denosumab and ibandronate groups (63.4 vs 64.4 years, respectively). Mean age at menopause onset was lower in the denosumab group than in the ibandronate group; however, this difference is unlikely to be clinically relevant (47.8 vs 48.9 years, respectively; *p* = 0.005).

**Table 1:** Baseline patient characteristics

	Denosumab (SC Q6M) (*n* = 224)	Ibandronate (oral QM) (*n* = 217)
Age, years		
Mean (SD)	63.4 (7.74)	64.4 (8.27)
Age at menopause onset, years		
Mean (SD)	47.8 (4.36)	48.9 (3.65)
Previous fragility fracture		
*n* (%)	57 (25.4)	38 (17.5)
Location of previous fragility fracture, *n* (%)		
*N*	57	38
Hip	7 (12.3)	0 (0)
Vertebrae	42 (73.7)	31 (81.6)
Other	9 (15.8)	7 (18.4)
Previous PMO therapy		
*n* (%)	44 (19.6)	26 (12.0)
Calcium/vitamin D supplements, *n* (%)		
Calcium plus vitamin D	116 (51.8)	115 (53.0)
Calcium or vitamin D	135 (60.3)	135 (62.2)
None	89 (39.7)	82 (37.8)
Systemic glucocorticoid use, *n* (%)		
Yes	11 (4.9)	5 (2.3)
No	213 (95.1)	157 (72.4)
NA	–	55 (25.4)
Secondary osteoporosis		
*n* (%)	13 (5.8)	9 (4.2)

*NA* not applicable, *PMO* postmenopausal osteoporosis, *QM* every month, *Q6M* every 6 months, *SC* subcutaneous, *SD* standard deviation

At enrolment, a significantly higher proportion of women in the denosumab group than in the ibandronate group had a previous fragility fracture (25.4 vs 17.5%, respectively; *p* = 0.0427). The incidence of previous hip fracture was higher in the denosumab group (12%) than in the ibandronate group (0%). For both the denosumab and the ibandronate groups, the most frequent location of previous fragility fracture was the vertebrae (73.7 and 81.6% of all fractures, respectively). The proportion of women with previous exposure to postmenopausal osteoporosis therapy was greater in the denosumab group than in the ibandronate group (19.6 vs 12.0%, respectively; *p* = 0.028). The proportion of women who used systemic glucocorticoids was 4.9% in the denosumab group and 2.3% in the ibandronate group.

### Treatment discontinuation

**Fig. 1:**
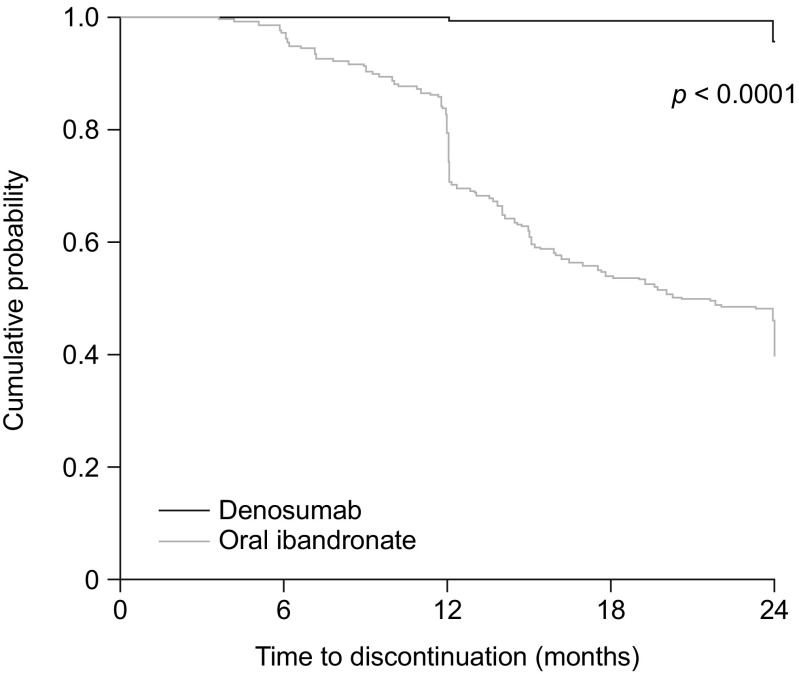
Kaplan–Meier plot showing the probability of patients continuing with treatment at a given time point

During the 24-month follow-up period, 10 women (4.5%) in the denosumab group and 122 women (56.2%) in the ibandronate group discontinued treatment. The median time between treatment initiation and treatment discontinuation was significantly longer in the denosumab group (729 days; interquartile range (IQR), 728.3–729.0) than in the ibandronate group (367 days; IQR, 354.0–484.8; *p* < 0.001). The cumulative probability of patients continuing with treatment at a given time point is shown in Fig. 1. Of the 10 women who discontinued denosumab, two did so for financial reasons and eight for unknown reasons. Most patients (80%; *n* = 8) who discontinued denosumab did not receive any further medication (Table 2). The main reasons recorded for discontinuing ibandronate (*n* = 122) were poor compliance (*n* = 42; 34.4%), financial reasons (*n* = 23; 18.9%), lack of effect (*n* = 19; 15.6%), adverse events (*n* = 13; 10.7%) and unknown (*n* = 22; 18.0%). Half of patients who discontinued ibandronate (*n* = 61) did not receive any further medication, and over one-third received denosumab therapy (*n* = 42; 34%) (Table 2).

**Table 2:** First osteoporosis medication prescribed following discontinuation of denosumab or monthly oral ibandronate

Medication administered after first discontinuation^a^, *n* (%)	Denosumab group (*n* = 10)	Monthly oral ibandronate group (*n* = 122)
Denosumab	1 (10)	42 (34)
Ibandronate	1 (10)	13 (11)
Alendronate^b^	0 (0)	2 (2)
Risedronate^b^	0 (0)	4 (3)
No medication	8 (80)	61 (50)

^a^Patients were considered to have discontinued treatment if they had reported discontinuation to their specialist, and this was recorded on their case report form; the initiated treatment was changed to another medication by the specialist (from denosumab to oral bisphosphonates, or from oral bisphosphonates to denosumab); or the patient stopped receiving medication for more than 60 days (the prescription coverage period was taken into account, i.e. 6 months for denosumab)

^b^Administered weekly

The hazard ratio for discontinuation (denosumab vs ibandronate) from the multivariate Cox model was 0.035 (95% CI 0.018–0.068; *p* < 0.0001). The best model for discontinuation contained only the following variables: treatment group, calcium supplementation and vitamin D supplementation.

### Denosumab persistence

Denosumab persistence rates (using the 60-day permissible gap) were 100, 99.1 and 98.7% at 12, 18 and 24 months, respectively. Sensitivity analyses using 30- and 90-day permissible gaps showed consistent results (Table 3).

**Table 3:** Persistence with denosumab

Persistence with denosumab, *n* (%)	30-day permissible gap (*n* = 224)	60-day permissible gap (*n* = 224)	90-day permissible gap (*n* = 224)
12 months	224 (100)	224 (100)	224 (100)
18 months	222 (99.1)	222 (99.1)	222 (99.1)
24 months	221 (98.7)	221 (98.7)	221 (98.7)

### Bone mineral density after 24 months of follow-up

**Fig. 2:**
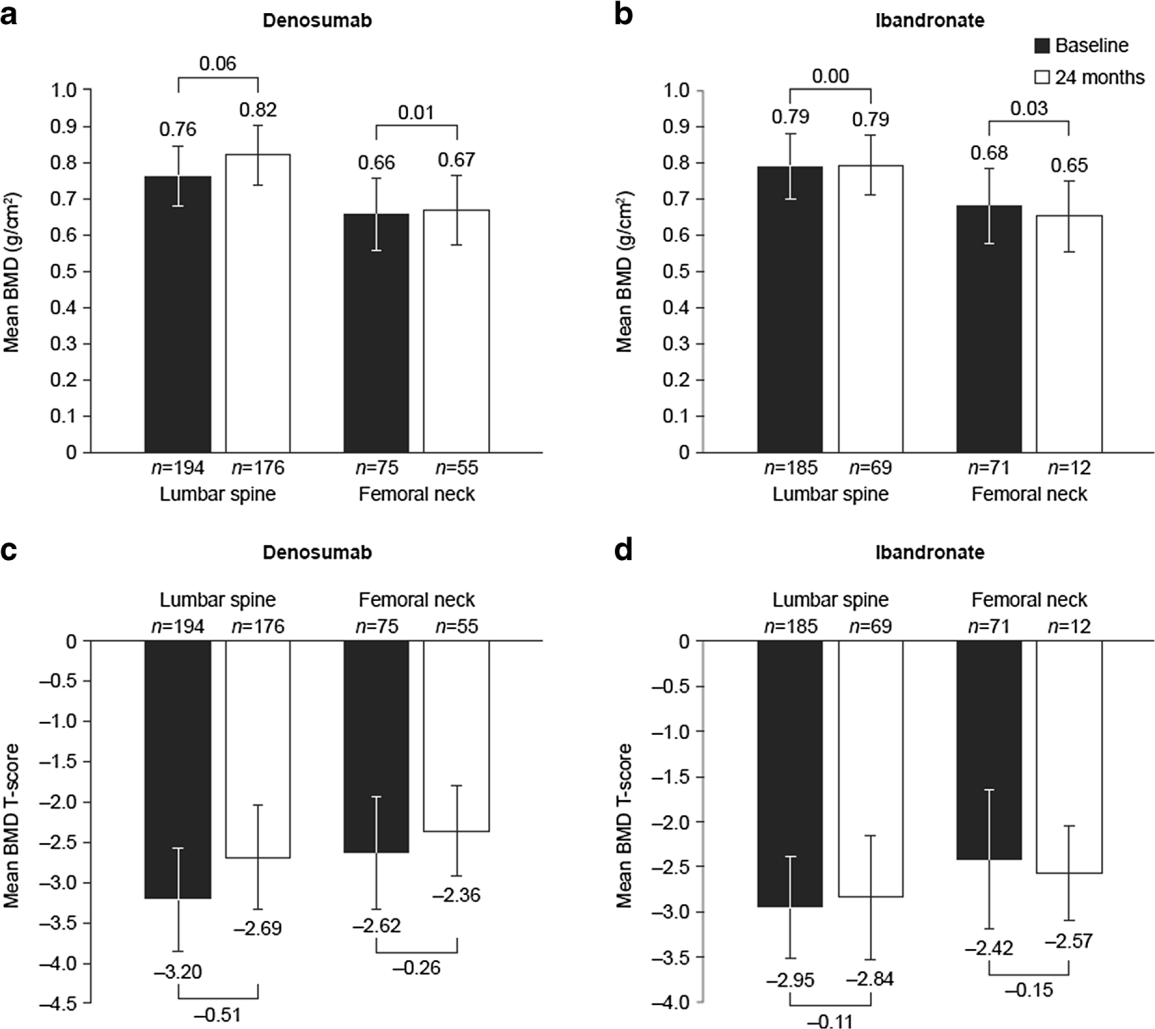
Mean BMD at baseline and 24 months for **a** denosumab and **b** monthly oral ibandronate and mean BMD *T*-score at baseline and 24 months for **c** denosumab and **d** monthly oral ibandronate. Error bars show standard deviation

**Fig. 3:**
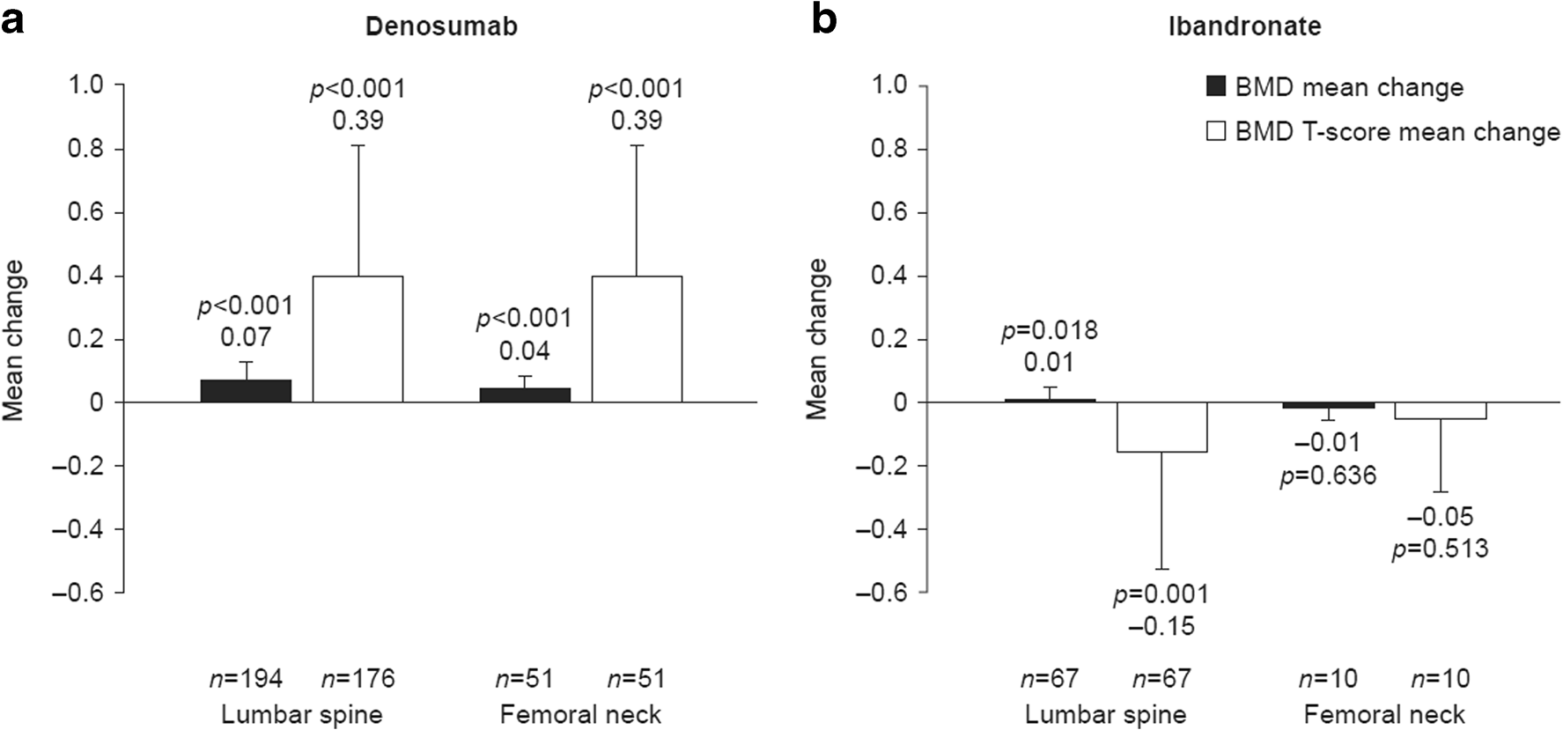
Mean change in BMD (g/cm^2^) and BMD T-score from baseline to 24 months for **a** denosumab and **b** monthly oral ibandronate. (The mean change in BMD and BMD *T*-score from baseline to 24 months of follow-up was calculated only for patients who had BMD and BMD T-score measurements from both time points.) Error bars show standard deviation. BMD, bone mineral density

Mean BMD and BMD *T*-scores at treatment initiation and at 24 months are presented in Fig. 2. The mean changes from baseline in BMD and BMD *T*-scores at 24 months are shown in Fig. 3.

At the lumbar spine, the percentage of patients eligible for BMD and BMD *T*-testing was similar in the denosumab and ibandronate groups (*n* = 194 (51.2%) and *n* = 185 (48.8%), respectively). Baseline mean BMD and BMD *T*-scores were slightly lower in the denosumab group than in the ibandronate group (Fig. 2). At 24 months, mean changes from baseline in BMD and BMD *T*-scores were statistically significant for denosumab (*p* < 0.001, for both) and ibandronate (mean change for BMD, *p* = 0.018; mean change for BMD T-score, *p* = 0.001) (Fig. 3). In post hoc exploratory analyses comparing BMD score between treatment groups after 24 months of follow-up, mean changes from baseline in BMD and BMD *T*-scores were significantly greater in the denosumab group than in the ibandronate group (*p* < 0.0001, for both).

At the femoral neck, the percentage of patients eligible for BMD and BMD *T*-testing was similar in the denosumab and ibandronate groups (*n* = 75 (51.4%) and *n* = 71 (48.6%), respectively). Baseline mean BMD and BMD *T*-scores were slightly lower in the denosumab group than in the ibandronate group (Fig. 2). At 24 months, mean changes from baseline in BMD and BMD T-scores were statistically significant for denosumab only (*p* < 0.0001, for both) (Fig. 3). Exploratory analyses found that mean changes in BMD and BMD *T*-scores from baseline were significantly greater in the denosumab group than in the ibandronate group (*p* = 0.004 and *p* < 0.001, respectively).

Overall, based on the highest BMD T-score recorded for each patient at each skeletal site, at 24 months, more patients in the denosumab group than in the ibandronate group achieved a BMD *T*-score higher than − 2.5 SD at all tested skeletal locations (lumbar spine, femoral neck and total hip; individual data are not presented for total hip owing to the low number of patients who had follow-up measurements recorded at this location) (46.9% [*n* = 105] vs 28.2% [*n* = 37]; *p* < 0.001).

### Fractures during 24 months of follow-up

During follow-up, three women (1.3%) receiving denosumab and eight women (3.7%) receiving ibandronate experienced one or more fragility fracture (*p* = 0.137). Fracture locations were recorded as hip, vertebrae or other. In the denosumab group, fractures occurred at the hip (*n* = 1; 33.3%), vertebrae (*n* = 1; 33.3%) or other location (*n* = 1; 33.3%). In the ibandronate group, fractures occurred at the hip (*n* = 7; 87.5%) or other location (*n* = 1; 12.5%).

## Discussion

To the best of our knowledge, this is the first retrospective, multicentre study examining medication-taking behaviour in women with postmenopausal osteoporosis treated with denosumab or a monthly oral bisphosphonate in real-world clinical practice in Bulgaria. Our data show that, over a 24-month observational period, fewer patients discontinued denosumab than discontinued ibandronate, and persistence with denosumab was high. Compared with ibandronate, treatment with denosumab was associated with significantly greater improvements in BMD *T*-scores at the femoral neck and lumbar spine. There were several notable differences in the baseline characteristics between the denosumab and ibandronate groups: a higher proportion of patients initiating denosumab had one or more previous fragility fracture or past exposure to postmenopausal osteoporosis therapy, baseline lumbar spine BMD and BMD *T*-scores were lower in patients treated with denosumab. These data suggest that patients initiating denosumab had a higher fracture risk than those initiating ibandronate and likely reflect real-world practice, whereby denosumab is prescribed to patients with severe osteoporosis, and commonly after suboptimal outcomes following bisphosphonate therapy. Although further studies are needed to determine whether this is indeed the case and which other factors may influence prescribing patterns, real-world studies in other European countries suggest that the favourable efficacy profile of denosumab may partly explain these behaviours. For example, in a recent prospective study evaluating the medication-taking behaviour of women with osteoporosis receiving denosumab in Germany, Austria, Greece and Belgium, the most common reason physicians gave for prescribing denosumab was that their patients had multiple risk factors for fracture or a history of osteoporotic fracture. Patients were also frequently prescribed denosumab owing to failure of, or intolerance to, their previous osteoporosis therapy [15].

This 24-month study demonstrated that few patients discontinue denosumab in real-world clinical practice in Bulgaria; only 4.5% of women initiating denosumab discontinued treatment within 24 months, compared with more than half of those initiating ibandronate. These data are in line with previous studies, which have shown that approximately half of patients discontinue oral bisphosphonate treatment within 12–24 months after initiation [8, 9]. The time to discontinuation for women treated with denosumab was also significantly longer than for those treated with ibandronate (median, 729 vs 367 days; *p* < 0.001). These data should, however, be interpreted with caution owing to the low number of patients discontinuing denosumab. Of the 10 women who discontinued denosumab, eight stopped therapy for unknown reasons and two did so for financial reasons. The main reason for discontinuation of ibandronate was poor compliance (34%); this is consistent with previous studies that have reported low compliance to bisphosphonates [20, 25]. The monthly oral administration of ibandronate is possibly a barrier to achieving compliance to therapy. Previous studies have shown that compliance to oral bisphosphonates is low owing to patient dissatisfaction with the route of administration and dosing frequency [20, 25]. Other reasons for patients discontinuing ibandronate therapy in this study were financial reasons (19%), lack of effect (16%) and adverse events (11%).

Over one third of patients who discontinued ibandronate switched to denosumab. Medication switching in chronic diseases is well documented [26]. Although the decision to switch from ibandronate to denosumab likely reflects the aforementioned reasons for discontinuation the impact of physician bias to switch from an older agent (ibandronate) to a newer agent (denosumab) cannot be ruled out. It should be noted, however, that the Bulgarian reimbursement committee require a medical reason, such as lack of effect of current medication, and consultation with a specialist endocrinologist or rheumatologist to support medication switching.

In this study, a high proportion of women were persistent with denosumab therapy; persistence was 100% at 12 months and exceeded 98% at 18 and 24 months. Numerous studies have reported on real-world persistence with denosumab [15, 16, 18, 19, 27]. In two prospective studies, one conducted in Europe by Hadji and colleagues and one conducted in North American by Silverman and co-workers, denosumab persistence ranged from 82 to 95% at 12 months and 75 to 86% at 24 months [15, 18, 19]. In a retrospective study by Karlsson and colleagues that analysed real-world data from the Swedish Prescribed Drug Register (*n* = 2315), denosumab persistence was 83% at 12 months and 62% at 24 months [16]. However, compared to the aforementioned studies, a retrospective study by Fuksa and colleagues that analysed data from the General Health Insurance Company of the Czech Republic (*n* = 7904) reported lower persistence rates for denosumab; persistence was 59% at 12 months and 35% at 24 months [27]. The differences in reported persistence between these studies may reflect the use of different definitions of persistence. In the studies by Hadji, Silverman and Karlsson, persistence at 12 months was defined as patients having received their second dose of denosumab within 6 months plus 56 days of their previous dose [15, 18]. Whereas, Fuksa and colleagues defined 12-month persistence as patients having received their third dose of denosumab within 6 months and 30 days of their previous dose [27]. Persistence with denosumab in our real-world study was higher than that previously reported in the literature. This may reflect the specialist physicians who participated in our study who were likely to provide good patient education, which has been shown to increase persistence [28].

While persistence with ibandronate could not be calculated in this study, persistence with oral bisphosphonates in the real-world setting has been shown to be low. In a meta-analysis of 40 retrospective studies assessing persistence with oral bisphosphonates, estimates of 24-month persistence ranged from 16 to 46%, with a pooled estimate of 30% [16]. It must be noted that most of these studies examined persistence of either daily or weekly oral bisphosphonates, rather than those with monthly dosing. Studies investigating the differences between oral bisphosphonates regimens have reported that monthly administration is associated with higher persistence that daily or weekly administration [29, 30]. In a randomised open-label multicentre study of persistence with osteoporosis medication, 6-month persistence was 57 and 39% with monthly ibandronate and weekly alendronate, respectively [29]. In a retrospective study that analysed data from the CISA prescription database in Japan, 24-month persistence was significantly higher with monthly oral bisphosphonates than with daily or weekly bisphosphonates (*p* < 0.001) [30]. Real-world persistence with monthly bisphosphonate regimens have, however, been reported to be low. In a large, Hungarian, retrospective, cohort study conducted in 296,300 women with postmenopausal osteoporosis, among 16,862 women receiving monthly oral bisphosphonates, persistence was at 27% at 12 months and 10% at 24 months [17]. In the same study, 12- and 24-month persistence with 6-monthly parenteral therapies was higher (81 and 38%, respectively) than persistence with daily/weekly/monthly oral therapies (32 and 16%, respectively) [17]. Therefore, the short interval between doses of oral bisphosphonates is likely to account, in part, for the poor persistence observed with bisphosphonates compared with denosumab, which is administered at 6-month intervals.

The importance of persistence in increasing BMD and reducing fracture risk is well established [11, 22, 31, 32]. At 24 months of follow-up, we observed significantly greater improvements in lumbar spine and femoral neck BMD and BMD *T*-scores for denosumab compared with ibandronate. Consequently, more patients in the denosumab group achieved a BMD *T*-score higher than − 2.5 SD. While our study was not powered to test for statistically significant differences in BMD changes between denosumab and ibandronate, and this analysis was exploratory, the increase in BMD and BMD *T*-scores observed for denosumab support data from other observational studies [24, 33]. The beneficial effect of denosumab on BMD has also been demonstrated over longer follow-up periods in clinical trial settings. The phase 3 Fracture REduction Evaluation of Denosumab in Osteoporosis every 6 Months (FREEDOM) extension study reported cumulative 10-year increases of 21.6% at the lumbar spine and 9.1% for total hip BMD [34]. Sustained improvements in BMD with denosumab have also been shown in an 8-year, phase 2 study in which lumbar spine BMD was increased by 16.5% and total hip BMD by 6.8% [35]. Improvements in BMD with ibandronate have also been reported in clinical trial settings; in an extension of the Monthly Oral iBandronate In LadiEs (MOBILE) ibandronate study, BMD at the lumbar spine increased by 8.4% after 5 years of follow-up [36].

It has previously been shown that denosumab increases BMD significantly more than bisphosphonates. In post hoc analysis of patients at high fracture risk enrolled in two multicentre, open-label studies, denosumab was associated with greater increases in BMD at 12 months than monthly oral ibandronate or risedronate (femoral neck 1.8 vs 0.3%, lumbar spine 3.7 vs 1.4%, total hip 2.2 vs 0.8%, respectively; *p* < 0.0001 for all comparisons) [37]. Furthermore, in a phase 3, randomised, double-blind study, BMD increases were significantly greater with denosumab than with alendronate (lumbar spine 5.3 vs 4.2%, *p <* 0.0001; total hip 3.5 vs 2.6%, *p* < 0.0001; femoral neck 2.4 vs 1.8%, respectively; *p* = 0.0001) [38]. Denosumab has also been shown to provide greater improvements in BMD than the parenteral bisphosphonate, zoledronic acid. In a randomised, double-blind trial, 643 women with osteoporosis, previously treated with oral bisphosphonates, were randomised to receive SC denosumab every 6 months plus intravenous (IV) placebo once, or IV zoledronic acid once plus SC placebo every 6 months. At 12 months, changes in BMD from baseline were significantly greater with denosumab compared with zoledronic acid (lumbar spine 3.2 vs 1.1%, *p* < 0.0001; total hip 1.9 vs 0.6%, *p* < 0.0001; femoral neck 1.2 vs − 0.1%, *p* < 0.0001; one-third radius 0.6 vs 0.0%, *p* < 0.05) [39]. The significant increases in BMD and BMD *T*-scores reported with denosumab in our study may, in part, explain the high persistence rates that we observed for denosumab; indeed, in real-world settings, it has been shown that significant increases in BMD increase patient compliance to therapy [40]. Conversely, the high persistence rates for denosumab may, in part, account for the greater improvements in BMD seen with this agent compared with ibandronate. Although it was beyond the scope of this study to assess changes in BMD only in patients who were persistent with treatment, the results of such analyses would be of interest and are recommended for future studies.

There are several limitations to our study. BMD was not measured in all patients at all skeletal sites throughout follow-up, limiting the patient sample for which changes in BMD could be calculated. However, this reflects real-world clinical practice. This was a retrospective study and patients were not randomised; thus, any comparisons between groups should be made with caution, and the different baseline characteristics of the enrolled patients should be considered, in addition to the prescribing habits and preferences of the physicians. The differences in the baseline characteristics of the patients receiving denosumab and those receiving ibandronate may have influenced the rate of treatment discontinuation in each group. However, the differences in patient characteristics between the denosumab and ibandronate groups reflect the differing patient populations who are likely to receive these agents in real-world practice. Additionally, because this study was conducted in centres specialised in the treatment of osteoporosis, results may not be reflective of medication-taking behaviour of patients treated in general practice. Moreover, because denosumab was a new agent at the time the study was conducted, some physicians may have been biased towards switching patients in the ibandronate group to denosumab, thus inflating discontinuation rates for ibandronate. However, this is reflective of real-world clinical practice. The small patient number may limit the interpretation of the results. Finally, although the 24-month follow-up period is in line with several other studies assessing medication-taking behaviour in women with postmenopausal osteoporosis [17, 20, 41, 42], a longer follow-up would be valuable in understanding the long-term discontinuation rates with these agents.

## Conclusions

This is the first real-world chart review study of medication-taking behaviour in women with postmenopausal osteoporosis receiving denosumab or ibandronate in Bulgaria. In real-world clinical practice, denosumab is frequently prescribed to patients with severe osteoporosis who are at a high risk of fracture. This was reflected in our study. Denosumab treatment was associated with significantly greater increases in BMD when compared with ibandronate. In addition, very few patients receiving denosumab discontinued therapy compared with patients receiving ibandronate. At 24 months, persistence with denosumab exceeded 98%. Consistent with the greater BMD increases observed, the low discontinuation rate and high persistence among women initiating denosumab could lead to improved clinical outcomes, including fracture risk reduction.
